# Multimodal Biosensing of Foodborne Pathogens

**DOI:** 10.3390/ijms25115959

**Published:** 2024-05-29

**Authors:** Najeeb Ullah, Tracy Ann Bruce-Tagoe, George Adu Asamoah, Michael K. Danquah

**Affiliations:** Department of Chemical and Biomolecular Engineering, University of Tennessee, Knoxville, TN 37996, USA; lzn122@utk.edu (N.U.); pvd756@vols.utk.edu (T.A.B.-T.); gasamoah@vols.utk.edu (G.A.A.)

**Keywords:** electrochemical, optical, biosensing, multimodal biosensing, food safety, foodborne pathogens

## Abstract

Microbial foodborne pathogens present significant challenges to public health and the food industry, requiring rapid and accurate detection methods to prevent infections and ensure food safety. Conventional single biosensing techniques often exhibit limitations in terms of sensitivity, specificity, and rapidity. In response, there has been a growing interest in multimodal biosensing approaches that combine multiple sensing techniques to enhance the efficacy, accuracy, and precision in detecting these pathogens. This review investigates the current state of multimodal biosensing technologies and their potential applications within the food industry. Various multimodal biosensing platforms, such as opto-electrochemical, optical nanomaterial, multiple nanomaterial-based systems, hybrid biosensing microfluidics, and microfabrication techniques are discussed. The review provides an in-depth analysis of the advantages, challenges, and future prospects of multimodal biosensing for foodborne pathogens, emphasizing its transformative potential for food safety and public health. This comprehensive analysis aims to contribute to the development of innovative strategies for combating foodborne infections and ensuring the reliability of the global food supply chain.

## 1. Introduction

Infections associated with microbial foodborne pathogens are enormous worldwide health challenges, affecting millions of people annually. The WHO (World Health Organization) estimates that nearly 1 in 10 individuals worldwide become infected after consuming contaminated food, leading to 600 million cases of foodborne infections annually [1]. The consequences extend beyond mere infection, with economic losses, hospitalizations, and even fatalities, imposing a heavy toll on societies. Pathogens like *Salmonella*, *Listeria monocytogenes*, *Escherichia coli*, and norovirus are responsible for numerous infections, highlighting the need for cautious monitoring and robust detection strategies. Efforts to control the impact of foodborne diseases are challenged by several factors, including the globalization of food trade, changes in food consumption habits, and the appearance of novel and infectious pathogens strains. These dynamics have heightened the complexity of protecting our food supply and emphasized the importance of adopting innovative technologies that can rapidly and accurately detect the presence of harmful pathogens [1,2].

Single-mode biosensors have limitations that make them less versatile compared to multimodal biosensors. One of the main disadvantages of single-mode biosensors is their restricted capability to detect multiple analytes simultaneously, limiting their versatility and efficiency [3]. Single-mode biosensors lack the sensitivity and specificity required for complex detection tasks, especially in situations where multiple targets need to be identified. Additionally, single-mode biosensors struggle with issues related to lower sensitivity and discrimination ability, particularly when faced with single-base mismatches or complex biological samples [4]. In contrast, using a multimodal approach in biosensing, particularly for a single target, is a significant improvement. Multimodal biosensors offer enhanced performance and reliability by integrating multiple sensing modalities, which can lead to improved accuracy and sensitivity in detecting specific targets. By combining different detection mechanisms, multimodal biosensors provide a more comprehensive analysis of biological samples, allowing for a more thorough and precise detection of single targets. For example, a multimodal biosensor coupled with electrochromic, photoelectrochemical, and spectral signals was developed for the sensitive visual identification of nonylphenol, showing the advantages of combining multiple detection modes for enhanced detection capabilities [5,6,7]. Another example is using a dual-readout biosensor for detecting nucleic acids and non-nucleic acids utilizing a CRISPR-ALP tandem analysis, demonstrating the versatility and efficiency of multimodal biosensors in detecting single targets with different characteristics [5,7,8]. Furthermore, by integrating different sensing techniques, such as immunological approaches, nucleic acid-based procedures, and biosensor-based techniques, multimodal biosensing can enhance the sensitivity, specificity, and rapidity of the detection of pathogens [9,10,11,12]. As an example, the combination of nucleic acid-based methods with biosensor-based methods provides rapid and accurate detection by targeting specific genetic sequences of pathogens and detecting their presence using highly sensitive biosensors [4]. In addition, the use of topological preservation methods and innovative biosensing techniques can contribute to the advancement of multimodal biosensing, enabling the identification of a varied range of compounds in a multi-mode system [13,14,15,16]. This approach can improve the overall performance of foodborne pathogen detection systems and enable real-time monitoring of food safety.

This article offers a thorough exploration of the current state of multimodal biosensing opportunities for foodborne pathogen detection. The focus will be on combined approaches that involve combining two or more biosensing techniques to enhance accuracy, efficacy, and precision. Various aspects of multimodal biosensing are covered, including opto-electrochemical, optical nanomaterial, multiple nanomaterial-based systems, hybrid biosensing microfluidics, and microfabrication techniques. It discusses the advantages and challenges, the potential applications of optical and electrochemical techniques, nanomaterials, and microfluidics, as well as their implications for food safety and public health.

## 2. Multiple Signal Channels

### 2.1. Fusion of Optical and Electrochemical Techniques

Multiple signal channel biosensing platforms represent a powerful approach by combining different sensing techniques, such as optical and electrochemical methods, to provide comprehensive and accurate detection of analytes. These platforms have gained significant attention due to their potential applications in numerous fields. Electrochemical biosensors are among the most widely used and successfully commercialized types of biosensors. They are popular because of their high sensitivity, selectivity, and simplicity. Enzyme-based electrochemical biosensors, in particular, have garnered increasing interest due to their promising applications in various areas. These biosensors use enzymes as recognition elements to selectively detect target analytes such as glucose, hydrogen peroxide, phenol, and cholesterol [16]. On the other hand, optical biosensors offer benefits including label-free detection, real-time detection, and enhanced sensitivity. They depend on the interaction between light and the target analyte to generate a signal for detection. Optical biosensing platforms can be based on numerous principles, comprising fluorescence, surface-enhanced resonance (SPR), and reflectivity measurements [17,18]. Supramolecular self-assembly is another approach for enhancing biosensing platforms. This technique has been used in enzyme sensing, providing a label-free biosensing system with excellent sensitivity and multimodal readouts. The ease of this process eliminates the need for costly substrate preparation and allows for naked-eye detection [19].

**Figure 1:**
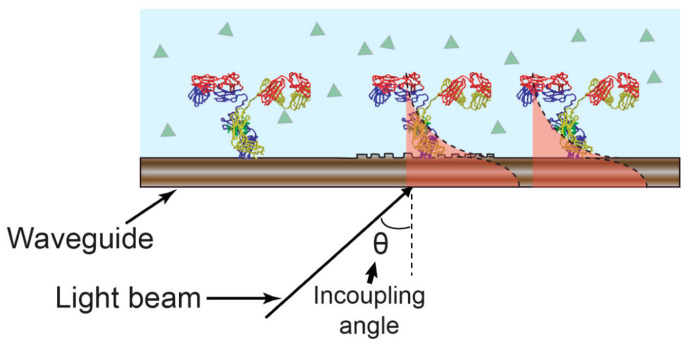
Sensing mechanism of OWLS. An incoupling angle variation in response to changes in refractive index affects the guided optical mode [20]. Reproduced with permission from José Juan-Colás et al. (2017), ©MDPI, 2014 (open access).

Electrochemical optical waveguide light mode spectroscopy couples evanescent field optical sensing with electrochemical regulation of surface adsorption procedures. A functional electrochemical is introduced by incorporating a layer that is optically and electrochemically compatible atop an optical formation. Optical waveguide light mode spectroscopy (so-called OWLS) serves as a label-free procedure for investigating the adhesion, adsorption, desorption, and bio-specific attachment processes on biomaterial surfaces, like Si(TiO_2_) [16,17]. In OWLS, a grating facilitates light coupling into a waveguide at specific angular values corresponding to transverse electric and transverse magnetic modes. These coupling angles change with a refractive index at the solution stage of a waveguide-solution connection. Observing these connecting angles allows us to determine the characteristics of an immobilized level [18]. Refer to Figure 1 for an illustration of the sensing mechanism.

**Figure 2:**
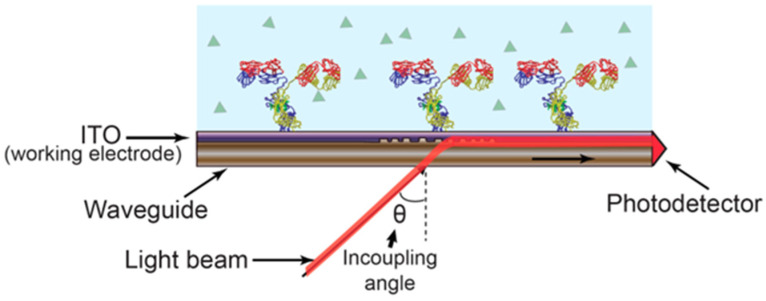
EC-OWLS, a technique combining electrochemistry with OWLS, operates dependent on a grating-coupled angle of light into a waveguide, influenced via the refractive index of molecules bound to the surface of a sensor. Simultaneously, the presence of ITO serves as an electrically charged layer, facilitating electrochemical processes [20]. Reproduced with permission from José Juan-Colás et al. (2017), ©MDPI, 2014 (open access).

The application of OWLS has been extended to monitor environmental pollution, lipid layer assembly, and protein–DNA interactions [21]. OWLS exhibits lower sensitivity compared to SPR due to the relatively inadequate light–matter contact at an interface, resulting in reported detection limits in a range of hundreds of ng/mL [22]. OWLS technology made its debut in a market with OWLS (Budapest, Hungary), launching an OWLS 210^®^ system in 2002. An integration of electrochemical analysis with OWLS was initially documented in 2002. Electrochemical capability was introduced by applying an indium tin oxide (ITO) thin layer as a working electrode on a waveguide surface. A flow cell was equipped with auxiliary and reference electrodes, establishing a 3-electrode electrochemical construction. The dual functionality of ITO, serving as both a conductive working electrode and a high refractive index waveguide, addresses both optical and electrochemical needs. Refs. [23,24] refer to Figure 2.

EC-OWLS was initially shown to monitor and regulate an adsorption polymer with an electrostatic interaction polymer based on the applied potential, offering insights into the kinetics of adsorption for charged moieties [23,24,25,26]. This method enables simultaneous control and in situ analysis of adsorption kinetics, tracking an adsorbed mass of charged molecules, and investigating the reversibility and irreversibility of an adsorption process. Its applications are diverse, including characterizing layer-by-layer gathering [27] of thin film polyelectrolyte multi-layers [28], assessing cell feasibility under applied currents [29], exploring surface variations [30,31], investigating molecular association [29], and evaluating a persistence of lactic acid bacteria [32]. Similar to EC-SPR, a procedure has instructed compatibility with EIS for analyzing lipid bilayer growth [33].

Multiple signal channel biosensing systems, combining optical and electrochemical techniques, possess immense potential for various applications, yet they face challenges requiring attention. One key issue is the optimization of signal transduction mechanisms to enhance sensitivity and specificity across diverse analytes. Future research could focus on developing novel materials and surface functionalization strategies to improve signal amplification and reduce non-specific interactions. Additionally, standardization of procedures and validation methods is crucial to ensure the reliability and reproducibility of results obtained from these platforms. Exploring miniaturization and integration with microfluidic systems could further enhance portability and on-site applicability, facilitating real-time monitoring in field settings. Advancements in data analytics and machine learning algorithms are also essential for efficient data processing and interpretation, enabling rapid and accurate detection of analytes.

### 2.2. Combination of Optical- and Nanomaterial-Based Methods

The combination of optical- and nanomaterial-based methods has demonstrated significant potential in various biosensing applications. Nanomaterials, such as transition-metal dichalcogenides (TMDs) and nanoporous anodic alumina (NAA), have been extensively explored for their unique properties and their ability to enhance biosensor performance [34,35]. TMDs, including MoS_2_, have gained interest due to their multi-dimensional structures and structure-dependent electronic, optical, and electrocatalytic properties. MoS_2_ nanostructures have found utility in both optical and electrochemical biosensing platforms. For example, 1D MoS_2_ quantum dots (QDs) have exhibited good photoluminescence, enabling optical biosensing of various analytes using a simple fluorometric technique. On the other hand, 2D MoS_2_ nanostructures have been investigated for electrochemical sensing due to their controllable electronic energy levels [34]. In addition, nanomaterials like gold nanoparticles and silica nanoparticles have also been widely employed in fluorescence-based biosensing. These nanomaterials offer superior optical properties, including brighter fluorescence, a broader range of excitation and emission wavelengths, and increased photostability compared to traditional organic dyes. They can be integrated into biosensing platforms to enhance sensitivity and enable a diverse selection of probes [36]. NAA is a versatile platform for optical biosensors. NAA possesses unique physio-chemical characteristics that make it suitable for designing biosensors in conjunction with various optical methods. It has been demonstrated that NAA can enhance the performance of optical biosensors and serve as an alternative to other nanoporous platforms. Researchers have explored the coupling of NAA with various optical detection techniques, and the performance of NAA-based biosensing devices has been evaluated [34,37]. Fluorescent sensing using carbon dots has also been advanced for food analysis, with common foodborne pathogens such as *E. coli*, *S. aureus*, *Salmonella*, and *Campylobacter jejuni* being targeted [38].

**Figure 3:**
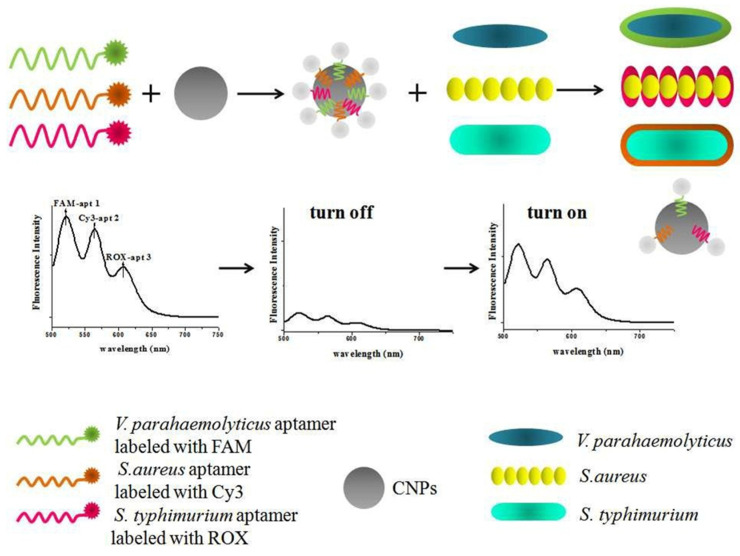
Schematic illustration of the multiplexed fluorescence resonance energy transfer from aptamer-modified dyes to carbon nanoparticles for the simultaneous detection of various pathogenic bacteria (*V. parahaemolyticus*, *S. aureus*, and *S. typhimurium*) [39]. Reproduced with permission from Duan et al. (2016) ©RSC, 2016.

For example, Figure 3 illustrates a multiplexed assay utilizing an aptamer-based platform, employing multicolor dyes as energy donors and carbon nanoparticles (CNPs) as the sole acceptor in fluorescence resonance energy transfer (FRET), enabling simultaneous detection of multiple bacteria (*V. parahaemolyticus*, *S. aureus*, and *S. typhimurium*). Specifically, fluorescein amidite (FAM)-apt 1, cyanine dye 3 (Cy3)-apt 2, and 6-carboxy-X-rhodamine (ROX)-apt 3 were employed as multicolor fluorescence probes for the recognition of specific targets. These aptamers bound to the surface of CNPs via π-π stacking interactions. Consequently, the energy donors and acceptors were brought into close proximity, resulting in the quenching of the fluorescence of the multicolor dyes. Upon introduction of the three bacteria (*V. parahaemolyticus*, *S. aureus*, and *S. typhimurium*), the aptamers exhibited a preference for binding to their respective pathogens. This binding event induced changes in the conformation of the aptamers, leading to the dissociation of the aptamer-labeled dye from the CNPs surface. The restoration of fluorescence intensity of FAM, Cy3, and ROX was directly correlated with the concentration of the three bacteria (*V. parahaemolyticus*, *S. aureus*, and *S. typhimurium*). This integrated approach offers a robust and sensitive means for the simultaneous detection of multiple bacterial species, with potential applications in biomedical diagnostics and environmental monitoring [39].

Optical- and nanomaterial-based methods offer viable paths for enhanced biosensor performance. However, challenges persist in optimizing the synergistic integration of these techniques to achieve maximal sensitivity and specificity. Future research directions could focus on developing novel nanomaterials with specific properties optimized for optical detection methods, thereby expanding the range of analytes and improving detection limits. Additionally, efforts should be directed toward improving fabrication techniques and assay procedures to ensure reproducibility and scalability across different biosensing platforms. Exploring advanced signal processing algorithms and machine learning techniques could further enhance data analysis and interpretation, enabling more robust and reliable biosensing applications in several fields, i.e., healthcare, environmental monitoring, and food safety.

### 2.3. Combination of Multiple Nanomaterials

The integration of multiple nanomaterials shows significant capacity in various biosensing applications. Nanomaterials bring unique properties that can enhance biosensor performance. For example, MXene-graphene nanohybrid thin film, fabricated through air-brush spray coating, serves as an efficient electrochemical biosensor for the identification of biomarkers. The integration of MXene and graphene enhances sensitivity, offering a promising platform for accurate biomarker detection [40]. GNP-adapted reduced graphene oxide nanosheets function as a dual-quencher system, enabling extremely sensitive recognition of carcinoembryonic antigens. This innovative approach enhances detection capabilities, emphasizing the potential for advanced and precise biomarker analysis [41,42].

**Figure 4:**
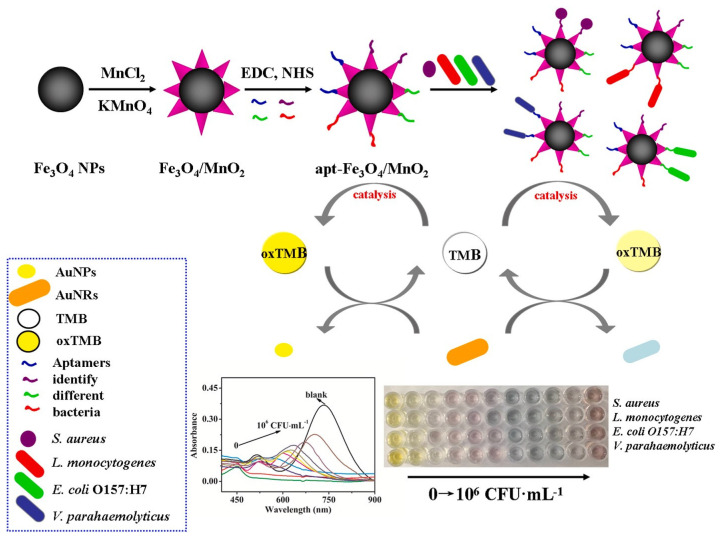
Schematic diagram of the multi-nanomaterials-based multi-colorimetric assay for the detection of foodborne pathogenic bacteria (*S. aureus*, *Listeria monocytogenes*, *E. coli* O157:H7, and *Vibrio parahaemolyticus*) [43]. Reproduced with permission from Zhang et al. (2021) ©Elsevier, 2021.

Figure 4 illustrates the synthesis process of Fe_3_O_4_/MnO_2_ nanomaterials with oxidase activity, along with the preparation of aptamer-Fe_3_O_4_/MnO_2_ probes for bacterial detection. Fe_3_O_4_ nanoparticles are synthesized via a hydrothermal method and subsequently coated with MnO_2_ nanoparticles through chemical oxidation, resulting in Fe_3_O_4_/MnO_2_ nanomaterials possessing oxidase-like activity. Four types of aptamer-Fe_3_O_4_/MnO_2_ probes are prepared by conjugating amino-modified bacteria-specific aptamers to Fe_3_O_4_/MnO_2_ nanomaterials activated with EDC and NHS via amide bond formation. Upon the introduction of different bacteria, the aptamer-Fe_3_O_4_/MnO_2_ nanomaterials selectively bind to specific bacterial surfaces, leading to a decrease in the oxidase activity of the probes due to the masking effect of bacteria on the catalytic active sites. Consequently, the oxidation of TMB (3,3′,5,5′-tetramethylbenzidine) catalyzed by aptamer–Fe_3_O_4_/MnO_2_ probes decreases. Gold nanorods (AuNRs) are subsequently etched by the oxidized TMB, resulting in polychromatic changes from yellow, yellowish, purple, blue, and indigo to brown. Additionally, the longitudinal displacement of AuNRs decreases with increasing concentrations of target bacteria. Based on this principle, a nanomaterials-integrated multicolor sensing system is established for the simultaneous qualitative and quantitative analysis of *S. aureus*, *Listeria monocytogenes*, *E. coli* O157:H7, and *Vibrio parahaemolyticus*. This innovative approach offers a promising strategy for sensitive and specific detection of multiple bacterial species in complex samples, with potential applications in food safety and clinical diagnostics [43].

Nanomaterials have emerged as pivotal components in sensing technologies, owing to their unique physicochemical properties and versatility. We acknowledge the significance of nanomaterials as a distinct category of sensing materials and the need to justify their inclusion within the framework of multimodal sensing. Our rationale is based on the ability of nanomaterials to serve as platforms for integrating multiple sensing modalities, including optical, electrochemical, and surface-enhanced Raman scattering, among others. Harnessing the synergy of the interactions between nanomaterials and different sensing mechanisms can achieve enhanced sensitivity, selectivity, and versatility in analyte detection in multimodal sensing. 

### 2.4. Hybrid Biosensing with Microfluidics

Hybrid biosensing with microfluidics has emerged as a favorable approach for various applications in biomedical research and diagnostics. These platforms combine the advantages of multiple sensing modalities, such as acoustofluidics, electromagnetic metamaterials, and fluorescence enhancement, with the capabilities of microfluidics for sample handling and manipulation.

**Figure 5:**
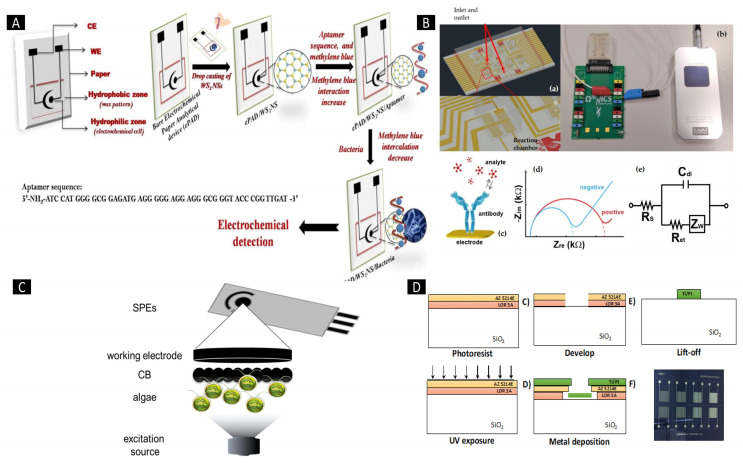
(**A**) Diagram illustrating a screen-printed paper-based aptasensor designed for *Listeria monocytogenes* detection. (**B**) Schematic depiction of a LOC device specifically designed for GLRaV-3 and GFLV sensing. (**C**) Illustration outlining a suggested algal/CB-SPE cytosensor scheme. (**D**) Process detailing the microfabrication of platinum interdigitated electrodes on a SiO_2_ wafer [50]. Reproduced with permission from Kulkarni et al. (2023), ©MDPI, 2014 (open access).

One example of such an integrated biosensing system is presented in the work of Zahertar et al. They propose a fully integrated biosensing platform that combines the functionalities of acoustofluidic technology and electromagnetic metamaterials into a single device. This platform enables microfluidic performance at acoustic frequencies and the detection of liquid characteristics at microwave frequencies, allowing for enhanced lab-on-a-chip devices [44]. Another example comes from Mishra et al. introducing an innovative paper-based aptamer-based electrochemical sensing device for *Listeria monocytogenes* detection, a known pathogen responsible for foodborne infections. This aptasensor offers numerous advantages: it is easy, consistent, disposable, and inexpensive (Figure 5A). The incorporation of aptamer enhances its capabilities, further contributing to the field of biosensors. This aptasensor’s sensing and quantification limits were established to be 10 and 4.5 CFU/mL, respectively, with a linear range of approximately 10^1^–10^8^ CFU/mL [45]. Buja et al. (2022) presented a microfluidics-based chip for ampelovirus and nepovirus detection. This chip features a multi-chamber design, enabling the simultaneous and rapid identification of these targets. It exhibits the ability to sense GLRaV-3 and grapevine fanleaf virus (GFLV) at dilution factors over 15 times higher compared to ELISA, thereby offering enhanced sensitivity in virus identification (Figure 5B). This microfluidic platform is characterized by its simplicity, speed, miniaturization, and cost-effectiveness, making it compatible with large-scale monitoring evaluations [46]. Antonacci et al. described an algal cytosensor designed for the electrochemical assessment of bacteria in wastewater using Chlamydomonas reinhardtii attached to carbon black (CB) nano-modified screen-printed microelectrodes. CB nanoparticles, owing to their ability to sense oxygen generated via algae and resulting current, served as nano-modifiers (Figure 5C). This sensor’s capability was assessed for the recognition of *E. coli* in real wastewater samples, exhibiting a linear concentration range from 100 to 2000 CFU/100 mL [47]. Sidhu et al. presented a microelectrode-based aptasensor on a platinum interdigitated electrode (IDE) for the identification of *Listeria* spp. in hydroponic growth media. This sensor was integrated into a hydroponic lettuce system for particle or sediment trapping, facilitating real-time evaluation of irrigation water (Figure 5D). Detailed electrochemical analysis was performed in the presence of *Listeria* spp. DNA ensued by calibration in various solutions. An aptasensor exhibited a 90% accuracy and might be reused several times after simple cleansing [48]. While some traditional methods for identifying foodborne pathogens are sensitive, their time-consuming nature limits practical application. Therefore, as Figure 5 demonstrates, developing novel techniques for the detection of foodborne pathogens is crucial. 

Microfluidics-based electrochemical biosensing has emerged as a promising approach for rapid pathogen detection through research and development efforts. Electrochemical biosensors utilizing aptamers or nucleic acids offer advantages, i.e., low detection limits and high sensitivity. However, enhancing their accuracy and stability remains a priority for further improvement [49].

Combining hybrid biosensing with microfluidics offers promising paths for biomedical research and diagnostics. However, challenges remain in optimizing these platforms for practical applications. Future research should focus on improving the sensitivity, specificity, and scalability of these integrated systems. Efforts to develop optimizing procedures and validation methods are essential to ensure the reliability and reproducibility of results across different settings. Collaborative interdisciplinary research is needed to explore novel materials and advanced signal processing techniques that can enhance the performance of hybrid biosensing with microfluidics. Moreover, the feasibility of deploying these platforms in real-world scenarios, particularly for foodborne pathogen detection, should be further investigated to address pressing public health concerns. Table 1 summarizes more applications of different biosensing techniques, taking into consideration the types of sensors, the limit of detection, the samples tested, and the targets. 

### 2.5. Integration of Nanoparticle-Based Screen Printing Technology

Nanoparticle and screen-printing technology show significant capacity in the detection and monitoring of foodborne pathogens. Researchers have explored innovative approaches to combat food contamination by utilizing the unique properties of nanoparticles, particularly in the context of food safety. The development of nanoparticle-based optical sensors and microarray technologies has enabled the simultaneous detection of multiple foodborne pathogens, thereby enhancing the efficiency and accuracy of pathogen identification [61]. These advancements in sensor technology, combined with nanoparticle applications, offer rapid and sensitive methods for detecting foodborne pathogens, crucial for ensuring food safety [62].

**Figure 6:**
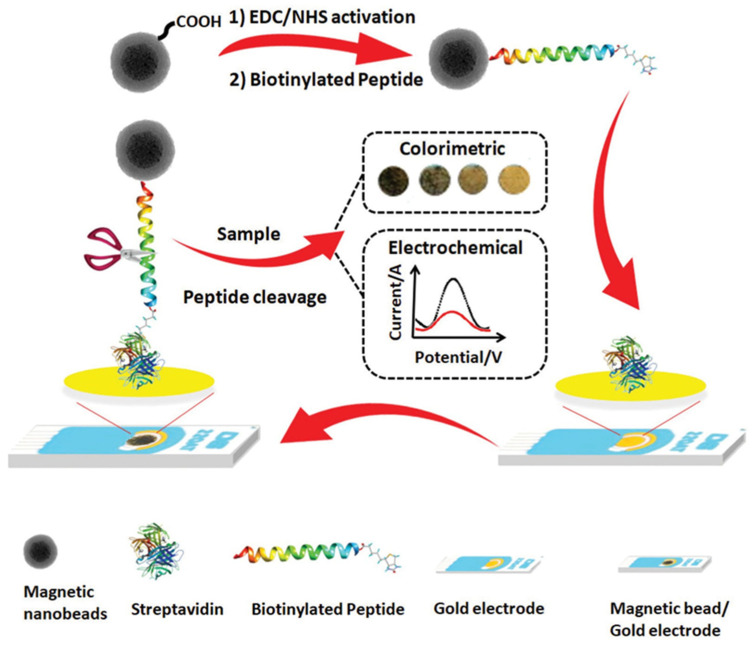
Illustration of a peptide/magnetic bead biosensor designed for *S. aureus* detection employing electrochemical and colorimetric methods on screen-printed gold electrodes fabrication [63]. Reproduced with permission from Eissa et al. (2020), ©Elsevier, 2020.

For example, as shown in Figure 6, a peptide-based biosensor was introduced for the detection of *S. aureus* using a gold screen-printed electrode as the transducer and a specific peptide sequence as the biorecognition element. This biosensor utilized both electrochemical and colorimetric detection approaches. The gold screen-printed electrode was modified with streptavidin, onto which a peptide sequence conjugated with magnetic nanoparticles was covalently immobilized as the biorecognition element. The peptide conjugated with magnetic beads was produced by activating carboxylated magnetic nanobeads and incubating them with the peptide. The presence of an Ahx linker in the peptide sequence prevented steric hindrance and facilitated access for protease cleavage at the desired site. The prepared biosensor could detect various concentrations of the analyte. Electrochemical measurements were conducted using the Square Wave Voltammetry (SWV) technique with ferro/ferricyanide as the redox marker. In the presence of the analyte, peptide cleavage occurred, leading to the release of magnetic nanoparticles from the transducer surface. This event enhanced the electron transfer rate between redox marker molecules and the transducer, resulting in increased SWV peak currents proportional to the analyte concentration. The signal-on biosensor demonstrated the ability to detect *S. aureus* in a linear range from 10^2^ to 10^8^ CFU/mL with a limit of detection of 3 CFU/mL [63]. 

## 3. Advantages and Challenges of Multimodal Biosensing

### 3.1. Enhanced Sensitivity and Specificity

Multimodal biosensing systems provide numerous advantages, including enhanced sensitivity and specificity in detecting various analytes, including foodborne pathogens.

Liu et al. emphasize that multimodal biosensing systems aim to achieve signal amplification, lower detection limits, and the ability to detect multiple targets. By combining different detection modalities, these platforms can enhance the specificity of detection, allowing for the sensing of analytes at lower concentrations. Specifically, they discuss the use of nanomaterial labeling in electrochemical immunosensors/immunoassays. The incorporation of nanomaterial labels, such as colloidal silver/gold, provides significant signal enhancement, enabling ultrasensitive electrochemical sensing of biothreat agents, infectious agents, and disease-related protein biomarkers [64]. Xu et al. discuss microfluidics and its capability to manipulate the size, shape, and composition of particles, resulting in the production of monodisperse particles with a remarkably limited range of sizes. This precise regulation allows for enhanced sensitivity in biosensing applications [65]. Siqueira et al. describe the incorporation of hybrid nanofilms on capacitive field-effect sensors, which can enhance the specificity of biosensors. Their study demonstrates that the incorporation of a hybrid urease-CNT nanofilm improves the output signal performance and sensitivity, enabling enhanced properties for urea detection [66]. Kim et al. mention the use of plasmon-enhanced fluorescence in biosensing for sensitivity improvement. This strategy utilizes plasmonic nanostructures to enhance fluorescence signals, leading to improved sensitivity in biosensing applications [67]. Xu et al. demonstrate the sensitivity enhancement achieved by using two-dimensional (2D) MXene in fiber optic biosensors. The metallic conductivity, high specific surface area, hydrophilic surface, and wide-band optical absorption of 2D MXene contribute to sensitivity enhancement in biosensing. They also support the sensitivity boost achieved by fiber optic biosensors through refractive index (RI) detection. Their study shows a significant sensitivity improvement in fiber optic RI sensors and fiber optic SPR sensors, which might be employed for the sensing of trace biochemical molecules [68].

Ullah et al. demonstrate the advantages of enhanced specificity and sensitivity in an electrochemical biosensor for the sensing of marine biological toxins [69,70]. Another study discusses how an electrochemical biosensor incorporating polycyclic aromatic hydrocarbons (PAHs) as modifiers achieves enhanced sensitivity and specificity for the identification of saxitoxin [71]. Furthermore, it highlights the advantages of label-free detection using a field-effect device-based biosensor for saxitoxin, emphasizing the enhanced sensitivity and specificity achieved [69,72]. Yadav et al. discuss various plasmonic-based optical detection platforms, including SERS, SPR, and LSPR. These platforms offer excellent specificity, sensitivity, and ease of operation, making them advantageous for the early sensing of biomarkers of infectious diseases [73]. Zargartalebi et al. introduce a novel approach for reagentless biosensing using the COVID-19 virus as a model target. The study demonstrates the high sensitivity and rapid sensing achieved by integrating a coffee-ring phenomenon into a detection scheme, enabling target pre-concentration on a ring-shaped electrode [74]. Wang et al. comprehensively examined recent advancements in DNA-based electrochemical biosensors designed for detecting foodborne pathogenic bacteria, providing insights into their efficacy, significance, and prospects for combating this threat [75].

### 3.2. Improved Analytical Performance

Multimodal biosensing, driven by improved analytical performance, offers several advantages in the field of biosensing.

The integration of multiple modalities, such as microfluidics and biosensors, allows for enhanced analytical performance. Microfluidic systems provide precise control over fluid flow, enabling high-throughput processing, improved transport, and reduced sample and reagent volumes. This integration enhances sensitivity, accelerates the mixing of reagents, and enables the simultaneous analysis of multiple analytes on a single platform [76]. Multimodal biosensing platforms enable multiplexed detection of multiple analytes. For example, Pappa et al. discussed the integration of an organic transistor array with finger-powered microfluidics for conducting multi-analyte saliva experiments. This platform offers the potential for non-invasive, multiplexed, and personalized point-of-care diagnostics [77]. Sadabadi et al. reported a compact multi-analyte biosensing device consisting of an organic electrochemical transistor microarray (so-called OECT) combined with a microfluidic pumpless system. This platform allows for the quantitative monitoring of lactate, glucose, and cholesterol levels [76].

The incorporation of different transduction elements and biorecognition elements in multimodal biosensing platforms enhances the specificity of sensing. Pappa et al. discussed the use of a “blank electrode” in biosensing platforms to boost a signal-to-noise ratio and the sensitivity. By incorporating an additional OECT functionalized with an unspecific protein, background interference can be minimized, leading to improved specificity [77]. Furthermore, multimodal biosensing platforms offer the advantages of disposability, portability, real-time sensing, and exceptional accuracy. These platforms have the potential to revolutionize point-of-care diagnostics, ecological analysis, healthcare, and accuracy agriculture [78].

Future research directions should focus on refining microfluidic systems for high-throughput processing, enhancing multiplexed detection capabilities, and improving specificity through innovative transduction and biorecognition elements. Interdisciplinary collaboration is key to addressing these challenges and realizing the full potential of multimodal biosensing platforms for transformative impact in diverse fields.

### 3.3. Minimization of False Positives and Negatives

Multimodal biosensing, focused on minimizing false positives and negatives, provides several advantages in various applications. One of the key benefits is the integration of different sensing modalities, like magnetic and optical biosensing, into a single platform. This integration allows for the concurrent recognition of multiple analytes or the utilization of complementary techniques to enhance sensitivity and accuracy. For example, optical magnetic multimodal bio probes have been designed to overcome the limitations of single-modal probes, capitalizing on unique electronic structures and magnetic and optical characteristics of lanthanide ions [79]. Another advantage of multimodal biosensing is the potential for increased detection sensitivity and accuracy. By combining multiple sensing modalities, detection limits can be enhanced, enabling the identification of analytes at lower concentrations. This is particularly important in clinical diagnostics, where early detection of diseases or pathogens is crucial for effective treatment and prevention. Multimodal biosensing can also reduce false positive cases, improving the specificity of the detection [5].

Multimodal biosensing devices can provide additional benefits in terms of responsiveness to various microenvironments and external stimuli. This flexibility allows for the recognition of analytes in complex samples or challenging conditions, enhancing the robustness and reliability of the biosensing system. Additionally, the use of biomimetic enzyme-like biosensing platforms can further enhance the performance of multimodal biosensors. These platforms mimic the catalytic activity of natural enzymes and can provide enhanced sensitivity and selectivity in detecting target analytes [80]. In the field of medical imaging, multimodal biosensing also offers advantages. For example, the integration of optical and magnetic imaging techniques allows for improved imaging depth and resolution. Optical imaging techniques, such as up-conversion nanoparticles, offer high spatial resolution but limited imaging depth, while magnetic resonance imaging (MRI) provides large penetration depth but lower resolution. By combining these modalities, the advantages of both techniques can be leveraged to achieve detailed imaging of internal structures with improved depth and resolution [79].

### 3.4. Challenges and Limitations

#### 3.4.1. Integration and Miniaturization

Integration and miniaturization are two key challenges in multimodal biosensing. The integration of different sensing modalities into a single platform requires careful consideration of the design and fabrication processes. This is significant for achieving portability, ease of use, and the ability to perform parallel, multiplexed, and automated studies. Integration also facilitates the incorporation of sensors and actuators with electronic experiment and measurement circuits, enabling seamless integration within a chip, package, or system [80]. However, integrating multiple modalities can be complex and may require advanced fabrication techniques and materials [81]. Miniaturization at the nanoscale is another challenge in multimodal biosensing. While miniaturization offers advantages like increased signal-to-noise ratio and improved sensitivity, there are trade-offs to consider. One trade-off is the longer time it takes to gather target analytes on the surface of a sensor owing to increased mass transport intervals. Careful consideration of signal transduction processes and reaction-transport kinetics is necessary to develop strategies that leverage miniaturization while maintaining minimal detection limits and immediate response times. Additionally, the choice of structure and device design, such as porous structures with a lower density of high pores, can impact the detection limit [80].

The challenges of integration and miniaturization in multimodal biosensing are not limited to technical aspects but also extend to commercialization and practical implementation. Commercialization of biosensors faces challenges such as sample preparation, introduction of nanomaterials, comparison to other technologies, and development of multiplex portable biosensors with low-cost formats. The stability of immobilized receptors and the fabrication of high-complexity components are also important considerations for commercialization [82]. Furthermore, the integration of microfluidics with multimodal biosensing systems presents additional challenges in terms of the design, miniaturization, and compatibility of wearable devices [83].

A comprehensive dual-mode lateral flow immunoassay is introduced, integrating colorimetric and photothermal capabilities through innovative bimetallic silver–gold urchin-identical hollow shapes. This approach not only demonstrates the sensitive exposure of *E. coli* O157-H7 but also emphasizes the significance of integration and miniaturization in advancing biosensing technologies [84]. The implementation of in situ fluorescent immunomagnetic multiplex recognition for foodborne pathogens in minimal quantities highlights significance in the domain of sensitive and specific pathogen detection, particularly for *E. coli* O157-H7 and *L. monocytogenes*. This innovative approach grounded in integration and miniaturization, shows promising implications for enhanced diagnostics [85]. A finger-actuated microfluidic sensor emphasizing colorimetric finding of foodborne pathogens, serves as a pivotal advancement in the field of miniaturized diagnostics, showing its potential for sensitive pathogen detection [86]. The significance of miniaturization and integration in the development of portable impedance immunosensing systems for the instant exposure of *S. typhimurium*, offering valuable insights into the potential for quick, sensitive, and portable recognition of this pathogen in various matrices [87]. The integration of masks/metallic-electrode compounds for silicon nano-sensor development is a critical area of research. The use of silicon as a substrate for biosensor arrays has been particularly prominent due to its ability to facilitate precise electrode design in micrometric dimensions and enable the integration of signal processing hardware components on the same substrate [88].

Integration and miniaturization in multimodal biosensing pose significant technical and practical challenges, requiring careful design considerations and advanced fabrication techniques. While promising enhanced functionality and efficiency, integration necessitates innovative approaches to balance the trade-offs associated with miniaturization, such as longer target analyte gathering times. Commercialization hurdles include sample preparation, nanomaterial introduction, and the development of low-cost multiplex portable biosensors. Stability of immobilized receptors and microfluidic integration with wearable devices are critical considerations. Future research should focus on streamlining fabrication techniques, enhancing receptor stability, and optimizing microfluidic designs through interdisciplinary collaboration. This collaborative effort is essential to advance multimodal biosensing toward practical implementation in various applications.

#### 3.4.2. Cost and Scalability

The cost and scalability of multimodal biosensing present significant challenges in their implementation and practical use. One challenge is the cost of the development and production of multimodal biosensing platforms. The integration of multiple sensing modalities often requires sophisticated and specialized equipment, materials, and fabrication techniques, which can be expensive. Additionally, the use of advanced technologies, such as nanomaterials and microfluidics, can further increase the cost of multimodal biosensing. The high cost of development and production can limit the accessibility and widespread adoption of multimodal biosensing technologies, particularly in resource-limited settings [82,89]. Scalability is another challenge in multimodal biosensing. As the number of sensing modalities or the complexity of the system increases, scalability becomes a critical consideration. Achieving scalability in multimodal biosensing requires efficient and scalable fabrication processes, as well as an ability to handle and analyze substantial volumes of data generated by multiple modalities [90,91]. Furthermore, the scalability of multimodal biosensing is closely linked to the commercialization and practical implementation of these technologies. The advancement of cost-effective and scalable manufacturing processes is essential for the widespread adoption of multimodal biosensing platforms [82,92]. In addition, the scalability of data analysis and interpretation methods is crucial for handling the large amounts of data generated by multiple modalities. Efficient algorithms and computational resources are needed to process and analyze the data in a timely manner, especially in real-time applications [90].

Multimodal biosensors for foodborne pathogen detection offer high sensitivity and specificity through the integration of multiple detection modalities, enabling a rapid and accurate analysis even in complex food matrices. However, complex design and fabrication processes, along with high production costs, pose challenges [91,93]. Opportunities lie in emerging technologies and the increasing severity of food safety regulations, which drive innovation and adoption. Integration with Internet of Things platforms enables real-time monitoring and quality control in food production and distribution [92,94]. Collaborative partnerships promote innovation and accelerate adoption. Competition, regulatory hurdles, and technological deterioration present threats to widespread adoption and commercialization. Despite challenges, the versatility and potential of multimodal biosensors drive ongoing research and development efforts in food safety [90,95].

## 4. Applications in Foodborne Pathogen Detection

Multimodal biosensing techniques have shown great potential in improving the detection of pathogenic microorganisms, i.e., *Salmonella*, *E. coli*, and *S. aureus* [96].

A multimode dot-filtration immunoassay (so-called MDFIA) for rapid *Salmonella typhimurium* detection was developed using MoS_2_@Au complexes. MoS_2_ served as a versatile antibody label, enhancing sensitivity through its intrinsic color, peroxidase-like activity, and photothermal effect. Immobilized on a nitrocellulose membrane, the immune–MoS_2_@Au complexes underwent visual qualitative analysis, showing color changes. The quantitative analysis utilized a complex’s photothermal effect, measuring temperature variation under laser irradiation. *Salmonella* levels were quantified by correlating temperature variation with a bacterial count logarithm. This method integrates visual and quantitative techniques, providing rapid and accurate biosensing potential [97]. Yu et al. presented a novel multimodal assay platform for *Salmonella* detection, a bacterium associated with foodborne diseases. A platform combines colorimetric, fluorescent, and magnetic nanospheres, offering a versatile approach to enhance sensitivity in detection. An integration of these diverse multimodal approaches provides a comprehensive and reliable method for *Salmonella* detection. A colorimetric aspect enables visual identification, a fluorescent component enhances detection sensitivity, and magnetic nanospheres facilitate the separation and concentration of a target pathogen. This innovative system possesses promise for rapid and effective *Salmonella* detection, with potential applications in food safety and public health [98]. Another study introduces a highly sensitive multimode dot-filtration strip for *Salmonella* identification. A system utilizes MoS_2_@Fe_3_O_4_ nanoparticles, indicating their efficiency in enhancing detection sensitivity. A multimodal approach integrates various modes of detection, offering a comprehensive and efficient method for *Salmonella* identification. A dot-filtration strip design enhances the practicality of an assay. This study presents a promising advancement in *Salmonella* identification methods, indicating a potential for rapid and sensitive diagnostics with significant applications in food safety and public health [99].

Another study presents a sensitive *E. coli* sensor featuring rapid response times, achieved through the immobilization of T4 bacteriophages on a multimode microfiber (MM). The MM, formed by tapering an optical fiber to micrometer dimensions under non-adiabatic conditions, allows for the excitation of fundamental and higher-order modes, creating mode interference primarily with HE11 and HE12 modes. By capturing *E. coli*, the immobilized T4 bacteriophage induces alterations in the refractive index of these modes. The sensor detects *E. coli* concentration by monitoring changes in a resonance wavelength of the mode interference spectrum, thereby offering a versatile and efficient method for *E. coli* detection [100]. Hu et al. present a novel detection platform designed for ultra-sensitive *E. coli* identification. This innovative system combines smartphone-readable colorimetry and inductively coupled plasma mass spectrometry (ICP-MS) to assist dual-mode detection. Notably, an integration of filter-assisted separation enhances the sensitivity of a platform. This study is highly significant as it directly addresses the development of a sensing module for *E. coli* recognition. The incorporation of smartphone-readable colorimetry and ICP-MS in a dual-mode setup represents a promising advancement, offering a potent approach for achieving ultrasensitive *E. coli* sensing [101]. In another study, optical fiber biosensors were employed for *E. coli* detection, a bacterium with potential bioterrorism applications and known for causing foodborne infections. A biosensor utilizes multi-mode optical fibers, with a sensing area enhanced by a 40 nm thickness Au-metal deposition to boost sensitivity. SPR is constructed, and it is observed that as a sensitive refractive index enhances, a resonant wavelength concurrently rises due to an associated reduction in energy. This research provides valuable insights into the development of optical fiber sensors for specific *E. coli* detection, addressing both health and security concerns associated with this hazardous bacterium [102].

Zheng et al. introduce a split-type multimodal biosensor for *S. aureus* detection. Utilizing FePor-TPA’s dual enzyme functioning and photocurrent response, the biosensor operates in two modes. In the first mode, the 2D FePor-TPA thin film exhibits catalase activity and sensitive photocurrent, decomposing H_2_O_2_ to O_2_. In the second mode, FePor-TPA exhibits excellent peroxidase activity, oxidizing TMB to oxTMB under acidic conditions. The biosensor offers a “signal-on” response, with increased targets generating more H_2_O_2_ and gluconic acid, enhancing sensitivity. The biosensor demonstrates high sensitivity and selectivity, making it a favorable tool for *S. aureus* exposure [103]. Zhang et al. developed a fluorescent aptasensor for sensitive *S. aureus* recognition. An aptasensor employs a dual-amplification technique by DNA-integrated walking and hybridization chain reaction (HCR). This innovative approach significantly enhances the sensitivity of *S. aureus* detection. A combination of DNA walking and HCR provides a robust and reliable amplification mechanism, contributing to the aptasensor’s overall performance. A fluorescence-based detection method offers a sensitive and specific means for the rapid identification of *S. aureus*, showing great applications in clinical diagnostics and food safety. This research presents a precious effort in the field of aptasensor development for pathogen detection [104]. In another investigation, a multimodal tracking approach was employed for controlled monitoring of *S. aureus* infections in mice. A study integrates various tracking modalities to comprehensively observe and analyze the progression of controlled infections. A multimodal approach enhances the precision and thoroughness of a tracking process, providing valuable insights into the dynamics of *S. aureus* infections in a murine model. This research offers a significant contribution to an understanding of infection dynamics and the development of more effective strategies for monitoring and managing *S. aureus* infections [105].

The studies demonstrate the efficacy of multimodal biosensing in detecting pathogenic microorganisms, like *E. coli*, *Salmonella*, and *S. aureus*, through various innovative techniques. However, challenges remain in optimizing sensitivity, specificity, and scalability for practical applications. Future research directions could focus on enhancing the integration of various sensing modalities to improve sensing accuracy and reliability further. Moreover, there is a need to investigate the feasibility of deploying these technologies in real-world scenarios, particularly in clinical diagnostics, food safety, and environmental monitoring, to address pressing public health challenges. Overall, continued innovation and collaboration are key to advancing multimodal biosensing for effective pathogen detection and disease management.

## 5. Conclusions and Future Perspectives

The convergence of various biosensing techniques into integrated multimodal platforms shows the promise to revolutionize foodborne pathogen detection. These advanced biosensors offer enhanced accuracy, efficacy, and precision, enabling earlier and more reliable identification of contaminants in food products. Through the synergistic combination of opto-electrochemical, optical nanomaterial, multiple nanomaterial-based systems, hybrid biosensing microfluidics, and microfabrication techniques, researchers and industries can address the limitations of single-mode biosensors and enhance the overall capabilities of pathogen detection. The integration of multiple biosensing strategies not only provides more comprehensive data about the target pathogens but also minimizes false-positive and false-negative findings. Despite the challenges of integrating different sensing principles, the benefits of improved performance and reliability justify the efforts required. As nanotechnology, microfluidics, and data analysis methods continue to advance, the potential for future multimodal biosensors to become an indispensable tool in ensuring food safety and public health is immense. With continuous research and collaboration, multimodal biosensing opportunities for foodborne pathogens will shape the state of biosensor technology, contributing to safer food products, reduced infections, and healthier communities. The journey towards these goals is marked by exciting advancements and a shared commitment to development innovation at the intersection of biosensing, nanotechnology, and public health.

The combination of multiple biosensing strategies to boost the accuracy, efficacy, and precision of foodborne pathogen detection shows a lot of potential in shaping the future of biosensor technology [80,81]. As research in multimodal biosensing for foodborne pathogens continues to progress, several compelling future perspectives emerge [90]. The field of nanotechnology is advancing rapidly, offering novel nanomaterials with enhanced properties for biosensing applications [82,89]. Future developments in engineered nanoparticles, nanowires, and nanocomposites are expected to further improve the sensitivity and selectivity of integrated biosensing platforms. Microfluidic technologies are enabling the miniaturization and integration of several processes on a single chip, enhancing the efficiency of sample preparation, detection, and analysis. The synergy between microfluidics and multimodal biosensing will enable rapid and precise pathogen detection, even with limited sample volumes. The fusion of data from multiple biosensing techniques will require advanced data analysis algorithms. Machine learning, artificial intelligence, and pattern recognition methods will play a significant role in extracting meaningful information from complex multimodal biosensor data, leading to more accurate and reliable results. Future multimodal biosensors may find applications beyond the laboratory, allowing real-time examination of food products throughout a supply chain. These biosensors could be integrated into portable devices for on-site detection, permitting rapid decision-making and intervention to prevent foodborne outbreaks. The development of modular and customizable biosensing platforms will empower researchers and industries to develop integrated biosensors to specific pathogen detection needs. Such platforms would allow the integration of different biosensing techniques based on the target pathogen and sample matrix. Incorporating these advancements and perspectives into multimodal biosensing for foodborne pathogens exhibits the significant potential to revolutionize food safety practices, ensuring the timely detection and prevention of foodborne infections.

## Figures and Tables

**Table 1:** Summary of different biosensing techniques for the detection of foodborne pathogens.

Pathogen	Sample	Detection Method	Analysis Time	LOD	References
*E. coli* O157-H7	Food samples	Surface plasmon resonance	30 min	50 CFU/mL	[51]
*E. coli*, *S. aureus*	Human blood	Fluorescence-based biosensors	120 min	4 CFU/mL	[52]
*S.typhimurium*	Food samples	Surface-enhanced Raman spectroscopy	Not Stated	35 CFU/mL	[53]
*Shiga toxin E. coli*	Water sample	Electrochemical impedance spectroscopy	1 h	10–102 CFU/mL	[54]
*Salmonella typhimurium*	Chicken sample	Magnetic biosensors	Not Stated	50 CFU/mL	[55]
*Salmonella*	Milk samples	MicrofluidicBiosensors	2 h	10 CFU/mL	[56]
*E. coli* O157-H7	Apple juice samples	Microfluidic-based immunoassay	1 h	10 CFU/mL	[57]
*E. coli*	Urine Sample	Colorimetric biosensors	Not Stated	35 CFU/mL	[58]
*E. coli*	Water sample	Anodic particle Colorimetry technique	Not Stated	1 CFU/mL	[59]
*Salmonella typhimurium*	Food samples	Luminescence bioassay method	Not Stated	10–15 CFU/mL	[60]
